# Health anxiety and attentional bias toward virus-related stimuli during the COVID-19 pandemic

**DOI:** 10.1038/s41598-020-73599-8

**Published:** 2020-10-05

**Authors:** Loreta Cannito, Adolfo Di Crosta, Rocco Palumbo, Irene Ceccato, Stefano Anzani, Pasquale La Malva, Riccardo Palumbo, Alberto Di Domenico

**Affiliations:** 1 Department of Neuroscience, Imaging and Clinical Sciences, University G. D’Annunzio of Chieti-Pescara, 66100 Chieti Scalo, Italy; 2 Department of Psychological Sciences, Health and Territory, University G. D’Annunzio of Chieti-Pescara, 66100 Chieti Scalo, Italy

**Keywords:** Psychology, Human behaviour

## Abstract

After the COVID-19 worldwide spread, evidence suggested a vast diffusion of negative consequences on people's mental health. Together with depression and sleep difficulties, anxiety symptoms seem to be the most diffused clinical outcome. The current contribution aimed to examine attentional bias for virus-related stimuli in people varying in their degree of health anxiety (HA). Consistent with previous literature, it was hypothesized that higher HA would predict attentional bias, tested using a visual dot-probe task, to virus-related stimuli. Participants were 132 Italian individuals that participated in the study during the lockdown phase in Italy. Results indicated that the HA level predicts attentional bias toward virus-related objects. This relationship is double mediated by the belief of contagion and by the consequences of contagion as assessed through a recent questionnaire developed to measure the fear for COVID-19. These findings are discussed in the context of cognitive-behavioral conceptualizations of anxiety suggesting a risk for a loop effect. Future research directions are outlined.

## Introduction

At the end of 2019, a new virus (known as COVID-19 or SARS-CoV-2) began threatening the physical health and lives of millions of people around the world, causing a lot of deaths after a few weeks. As reported by World Health Organization, the high contagiousness and rapid spread of COVID-19 induced a considerable degree of fear, worry, and concern in the general population, resulting in an overall worsening of individuals’ psychological health^1^. For this reason, the World Health Organization has issued guidelines for managing the problem taking into consideration both medical and psychological perspectives. Although several studies on COVID-19 focused on infection spread, containment measures, and potential vaccines, very little cognitive research has been conducted, so far, to address the impact of COVID-19 emergency on mental health and, therefore, possible treatment. According to a recent study on the Chinese population, which was the first in chronological order to manage the pandemic emergency, increased anxiety seems to be the most predominant symptom, followed by depression and sleep disorders^2^. Similar symptomatic clusters have been reported for the Italian population as well^3^. Several possible causes of this increased anxiety have been proposed, such as the impact of COVID-19 on academic life in the student population or on future employment and economic stability in general^4–6^. Furthermore, anxiety symptoms severity has been related to the absence of interpersonal relationship and loneliness, possibly attributed to the physical and social distancing imposed during the quarantine period^7,8^. Among other possible causes of increased anxiety levels, one of the most cited and shared is the fear of contagion, which led researchers to develop specific instruments to assess it^9,10^. These findings are not unexpected considering that fear is an evolutionary adaptive response to the presence of a dangerous and threatening stimulus that functionally motivates individuals to behave responsibly^11^. However, excessive fear could also be detrimental since it is linked to adverse mental outcomes^12^, such as anxiety itself, which can be defined as the fear of a dangerous stimulus when that stimulus is not present or very unlikely to occur. Fear conditioning indeed, is one of the main processes involved in anxiety pathogenesis^13^, suggesting the central role of fear of contagion on anxiety. Thus, previous research on individuals with anxiety may be useful to contain and manage possible consequences on the population's mental condition, due to the pandemic emergency.

### Attentional bias and anxiety

From a cognitive perspective, there is a large body of research exploring the relationship between clinical anxiety and attentional modification. Specifically, the tendency to pay attention to threats, known as attentional bias (AB), refers to a higher level of attentional allocation to threatening stimuli compared to neutral ones^14–16^. Numerous studies highlighted that anxious individuals show an AB toward threatening information sources. This effect is less consistent and generally not observed in non-anxious individuals^17^. Among anxious populations, AB toward threats is a robust phenomenon^14,18^ observed with specific neural connectivity subtypes^19^, across several different experimental tasks, and different anxiety disorders. For example, in a dot-probe task, individuals with a generalized anxiety disorder (GAD) show faster reaction times toward probes that replace threatening faces compared to probes that replace neutral faces^20^. Similarly, an AB toward health-threat related stimuli has been proposed as the primary pathogenetic factor for the development and maintenance of health anxiety (HA), previously termed “hypochondriasis”. Notably, patients with HA show steady alertness and hypervigilance for potential internal and external health-threat information^21^. Consequently, the attentional system of HA is assumed to be particularly sensitive to health-threat stimuli and biased in its favor^22,23^. Thus, to better understand the relationship between increased anxiety and fear of contagion among the population during the pandemic, the assessment of attentional bias toward virus-related stimuli is paramount. This study aims to investigate how, during the lockdown phase in Italy, HA levels were associated with an attentional bias toward virus-related stimuli and to what extent this relationship was mediated by specific fear of contagion for COVID-19.

## Results

As the first step, the correlations between WI-7 and Fear for COVID-19 questionnaire’s factors were computed (see Table 1, also presenting descriptive statistics). Significant correlations between general HA level (WI-7) and fear for COVID-19 were detected, as well as between the factors of the Fear for COVID-19 questionnaire (“belief of contagion” and “consequences of contagion”) (see Table 1). Then, to examine whether “belief of contagion” and “consequences of contagion” mediate the relationship between HA level and AB toward virus-related stimuli, we performed a serial mediation analysis using Model 6 in the PROCESS tool^24^, an SPSS macro for mediation, moderation, and conditional process modeling. A serial model was chosen since our mediators might causally influence each other, causing a violation of the assumption required for the parallel mediation model^25^. Specifically, the partial correlation between the two mediators, “belief of contagion” and “consequences of contagion” remains significant after controlling for HA (r = 0.44, p =  < 0.001), suggesting the possibility that one mediator affects the other, which is the focus of serial mediation. This conclusion, as suggested by the partial correlation results, can be easily explained from a theoretical perspective. Indeed, it is reasonable to hypothesize that different beliefs about contagion may affect the worry for possible consequences.

**Table 1:** Descriptive statistics and correlations for study variables. ***p < .001.

Variable	*n*	*M*	*SD*	1	2	3
1. WI-7	132	2.24	1.74	**–**	**–**	**–**
2. Belief of contagion	132	38.28	16.89	0.343***	**–**	**–**
3. Consequences of contagion	132	49.10	23.11	0.498***	.532***	**–**

**Figure 1:**
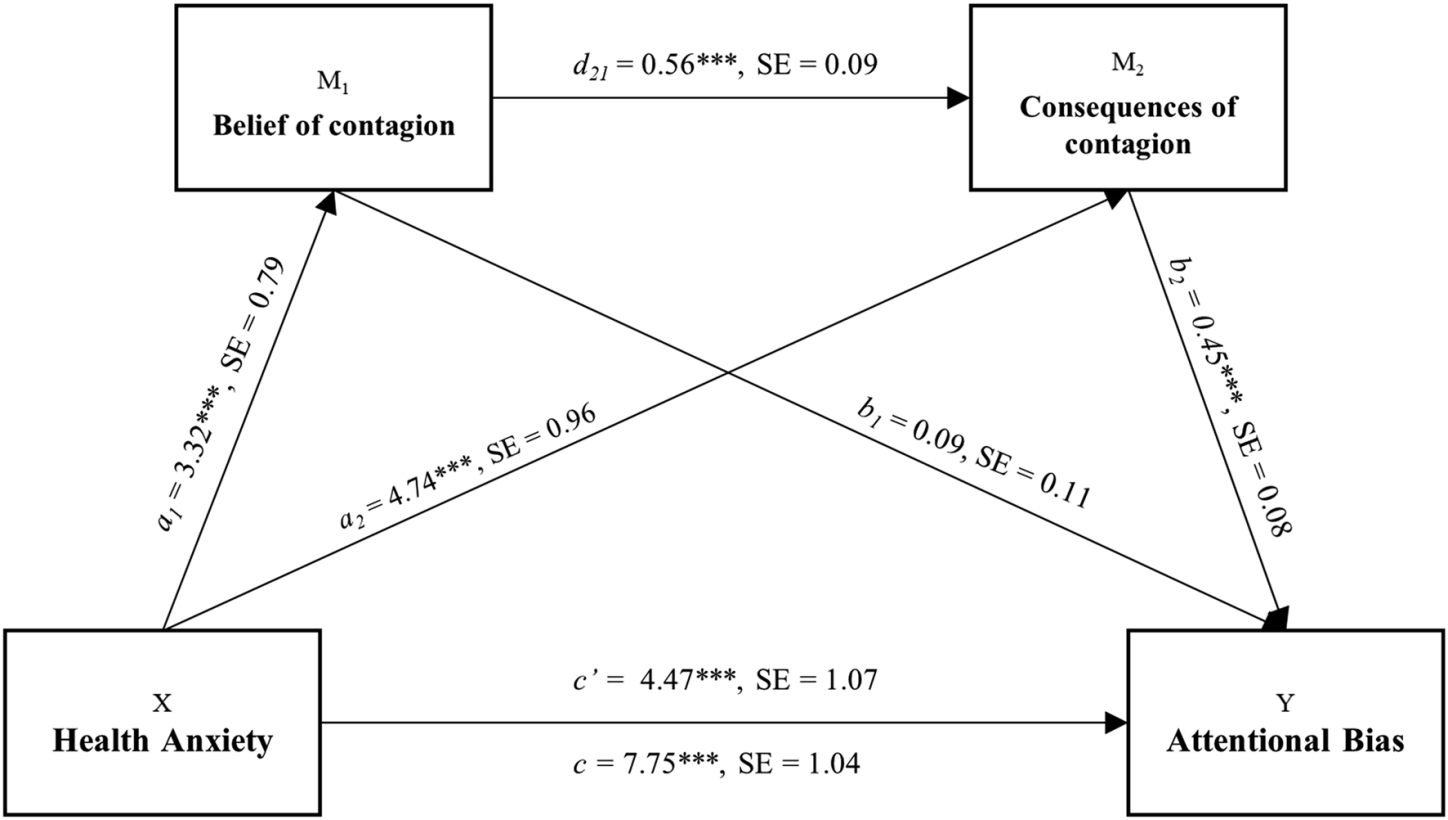
Serial mediation model. ***p < .001, *SE* standard error.

Serial mediation model permits the study of direct and indirect effects of X, the independent variable (HA) on Y, the dependent variable (AB) while modeling a process in which X causes M_1_ (belief of contagion) which, in turn, causes M_2_ (consequences), concluding with Y (AB) as an outcome. Thus, in this model, a mediator is assumed to have a direct effect on the other (path d_21_) and the independent variable (HA level) is expected to influence mediators in a serial way that ultimately influences the dependent variable (AB). As illustrated in Fig. 1, the total effect (path c), is the sum of direct (path c’) and indirect effects (path ab) on AB Specific indirect effects (paths a_1_b_1_ and a_2_b_2_) suggest the role of M_1_ (belief of contagion) and M_2_ (consequences of contagion) in the relationship between HA and AB respectively. At last, the overall influence of mediators is depicted by the total indirect effect (see Table 2). The model was run allowing bootstrapping with 5000 samples. Bias-corrected point estimates for the indirect effects of the HA on AB were calculated, together with standard errors and 95% confidence. A significant direct link between HA level and AB for virus-related stimuli (path c’) was detected, with higher HA level predicting stronger AB. Furthermore, results revealed that the HA level directly predicts “belief of contagion” (path a_1_) and that “belief of contagion” predicts “consequences of contagion” (path d_21_), which confirm the choice of a serial mediation model instead of a parallel one. Moreover, a significant direct relationship subsists between HA level and “consequences of contagion” (path a_2_), suggesting the validity of a model where “belief of contagion” acts as a mediator between general HA level and perceived “consequences of contagion” due to the specific COVID-19. The direct relationship between “belief of contagion” and AB (path b_1_) was not significant, while it was between “consequences of contagion” and AB (path b_2_). This relation indicates that a higher belief of contagion does not necessarily predict stronger AB. Notably, the double mediation indirect effect was significant, suggesting the existence of a causal chain that explains the relationship between health anxiety and attentional bias, passing through belief of contagion and consequences of contagion serially. Overall, the model explained 30% (R^2^ = 0.299) of variance in AB (see Table 2 for detailed results). These results suggest that, although individuals with general high HA levels are expected to show stronger AB, the belief of contagion and concerns about possible consequences of contagion due to COVID-19 double mediate this relationship.

**Table 2:** Serial mediation model predicting attentional bias (N = 132).

Path estimates	Coefficient (SE)	95% CI	
		LL	UL
a_1_	3.32 (.79)***	1.74	4.89
a_2_	4.74 (.96)***	2.83	2.65
d_21_	0.560 (.09)***	0.36	0.75
b_1_	0.091 (.11)	− 0.13	0.31
b_2_	0.450 (.08)***	0.27	0.62
c	4.47 (1.07)***	2.35	6.60
c’	7.75 (1.04)***	5.69	9.81

Indirect effects	Effect (SE)	95% CI	
		LL	UL
Total	**0.231**	**0.015**	**0.325**
HA → Belief of contagion → AB	0.021	− 0.024	0.073
HA → Consequences of contagion → AB	**0.150**	**0.085**	**0.237**
HA → Belief of contagion → Consequences of contagion → AB	**0.059**	**0.028**	**0.107**

Coefficient, non-standardized B coefficients

*SE* standard errors, *CI* bias-corrected and accelerated 95% confidence interval, *LL* lower limit, *UL* upper limit, *HA* health anxiety, *AB* attentional bias, 5,000 bootstrap samples.

Significant indirect effect in bold. ***Path coefficient significant at p < .001.

## Discussion

This study primarily aims to investigate the impact of health anxiety on attentional bias toward virus-related stimuli during the COVID-19 pandemic in the nonclinical population. Furthermore, we aimed to explore whether this relationship was mediated by specific beliefs related to the pandemic emergency. Attentional bias was measured via a visual dot-probe task that used both neutral and virus-related pictures of objects as stimuli. Participants’ reaction times were recorded to compute individual attentional bias to be associated with health anxiety levels measured with the WI-7. Moreover, participants completed the Fear for COVID-19 questionnaire allowing to obtain two independent factors: “belief of contagion” and “consequences of contagion”. Then, a double mediation model was performed by entering the two factors as serial mediators, the health anxiety’s level as the predictor, and the attentional bias score as the outcome. Results highlighted that health anxiety directly predicts attentional bias towards virus-related stimuli. At the same time, the model improved when considering the indirect effects of “belief of contagion” and “consequences of contagion”. However, “belief of contagion” did not seem to impact attentional bias directly but through the influence of “consequences of contagion”. In summary, the model suggested that higher health anxiety directly predicts higher attentional bias. The model highlighted, as well, an indirect path where higher health anxiety is associated with a stronger belief of contagion, which in turn, affects belief about future consequences of contagion, which finally predicts higher attentional bias towards virus-related stimuli.

These results are in line with previous findings on the relationship between attentional bias and expectancy bias. Indeed, theories such as the combined cognitive biases hypothesis suggest that cognitive biases (e.g., in expectancies and attention, but also in memory or interpretation) interact and mutually enforce each other^26^. For example, it has been widely shown how anxious and phobic individuals overestimate the likelihood of facing the threat, known as *encounter* expectancy bias, and the likelihood of incurring aversive consequences due to the encountered threat, known as *consequences* expectancy bias^27^. Similar findings have been reported on the relationship between expectancy biases and response to acute pain^28,29^. In the present work, the mediating variable “belief of contagion” refers to the probability of incurring in contagion, therefore it may be considered a measure of the encounter expectancy bias. The mediating variable “consequences of contagion”, instead, refers to the expected intensity of negative outcomes produced by the threat and it can be considered as a measure of the consequences expectancy bias. In this regard, the current results may contribute to the literature about expectancy biases in healthy population. Interestingly, in previous studies no encounter expectancy bias was reported among healthy individuals, but it was suggested that an a priori bias for consequences may exist for threats emanating from evolutionarily salient stimuli^26^. This evidence might be related to our results that highlighted how “belief of contagion” does not directly influence attentional bias but through the mediation of “consequences of contagion”.

Altogether, these results may be particularly useful to plan possible psychological interventions to counteract the effect of COVID-19 on population mental health. Indeed, while the evidence of a strong link between health anxiety and attentional bias in a nonclinical population (similar to the attentional pattern identified in the clinical population) could be reasonably considered worrying at first glance, it could also represent an opportunity to pave the way for future treatments^30^. Attentional bias associated with anxiety disorders has been the focus of several cognitive training, known as Attentional Bias Modification (ABM). Since its very early attempts of application, this training showed increasing evidence suggesting a significant effect of ABM on brain activity in the neural networks associated with the generation of affective states^31^. Among other clinical disorders, this training seems to be effective not just with anxiety disorders^32–34^ but also with specific phobia^35^. These findings appear to be particularly significant since the results of the current study suggest a joint effect of (nonspecific) health anxiety level and (specific) fear of COVID-19. Moreover, several attempts have been made to apply the ABM protocol remotely, in a web-based version^36^. This is especially relevant when considering the need to follow the social distancing guidelines during the lockdown period due to pandemics and the consequent difficulty in conducting research and clinical activities. Therefore, possible future directions may involve the development of a specific ABM intervention program for fear of COVID-19 to be administered via the web. A remote intervention would have the twofold advantage of reducing the costs of the intervention and overcome the logistic difficulties while guaranteeing monitoring and treatment services for the population's psychological well-being.

However, some limitations of the present results should be noted. The uncontrolled experimental setting should be considered as a potential limit. Given the exceptionality of this data collection, it was not possible to strictly control whether participants performed the task as requested. Moreover, since each participant completed the task on his/her personal computer, technical features (e.g., monitor refresh rate) were not controlled. We tried to limit the effect of some confounding factors, for instance requesting all participants to perform the task at the same time of the day (i.e., afternoon). Also, we would like to highlight that web-based data collection in the attentional bias field are increasingly common, suggesting the reliability of this online-remote method^37^. Hence, we are reasonably confident in our data’s reliability.

## Methods

### Participants

A total of 132 (91.7% female, mean age 24.4 ± 5.1 SD) Italian participants were enrolled in the study. Participants were recruited and provided written informed consent following the ethical standards of the Declaration of Helsinki and IRB approval was obtained by the University G. D’Annunzio institutional ethical committee. The experimental methods were carried out in accordance with the approved guidelines. Participants received no monetary or other forms of compensation for their participation. Since the study was carried out during the lockdown phase in Italy and due to the impossibility to invite participants in the laboratory, the whole procedure was conducted remotely. Participants were instructed to perform the task and answer questionnaires while being alone in a quiet room. For the visual dot-probe task, participants were asked to sit in front of a computer screen while maintaining a distance from the screen of about 60 cm for the entire duration of the task.

### Stimuli selection

A total of 60 pictures of objects, standardized for size and brightness, were selected from the web (for details see Supplementary Table S1 online). The task was administered through the recently released E-prime Go software, which allows remote data collection (pstnet.com). A subsample of participants (N = 86) were presented each object (1000 ms) in random order. After each object, participants were asked to rate on a scale from 0 (not at all) to 100 (extremely) how much the object just saw was related to the COVID-19. Based on the participants’ ratings, 40 objects were selected and divided into 2 categories. Thirty objects, rated as weakly associated to the virus (rating score ranging from 6 to 22.5), were attributed to the neutral stimuli category while the others 10 objects, rated as strongly associated to the virus (rating score ranging from 82.7 to 98.8), were attributed to the virus-related stimuli category.

### Visual dot-probe task

**Figure 2:**
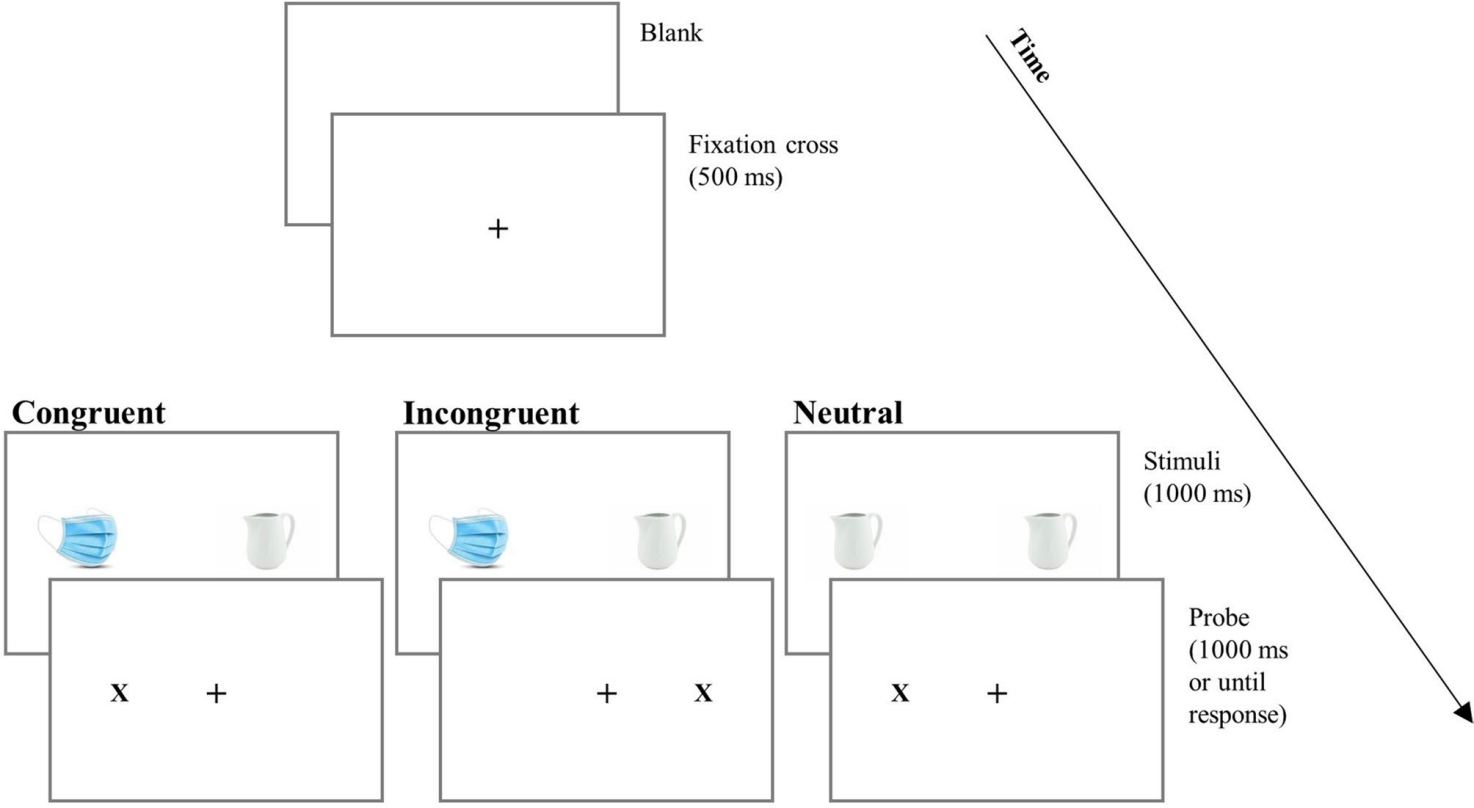
Trial structure and experimental conditions in the visual dot probe task.

Attentional bias was measured by a visual dot-probe task (see Fig. 2), using the Inquisit Web software’s script (millisecond.com). All data were collected during afternoon sessions, in the last week of April (from April 23rd to April 29th, 2020). On average, the experimental session lasted 5.6 min ± 2.6 SD. Based on Miller & Fillmore (2010), the script implemented a visual dot-probe procedure to measure attentional bias with images^38^. For a typical laptop screen size of 15.6 inches, pictures were presented in a box of 6 × 7 cm (visual angle = 5.72° × 6.67°, calculated using a viewing distance of 60 cm) to the left and right sides of fixation cross, with distance of 10 cm between the two. The 10 pairs of virus-related/neutral objects were presented 4 times based on the four possible object/probe combinations (the position of the object on the left or the right and the position of the probe on the left or the right), thus obtaining 40 test trials. Also, there were 40 filler trials, which consisted of 10 pairs of neutral images presented four times each. The filler trials are commonly included in tasks of attentional bias to reduce possible habituation to stimuli that might occur if all trials contained virus-related images. The 40 filler trials were intermixed randomly among the 40 test trials for a total of 80 trials. The task consisted of two blocks: the first block with 10 practice trials and the second block with the 80 task trials (40 test trials and 40 filler trials), randomly sampled without replacement. After the presentation of a fixation cross (+) in the center of the screen (500 ms), two side by side objects (virus-related and neutral objects for test trials, and both neutral objects for filler trials) were presented for 1000 ms.The position of the pictures was randomly chosen to be either on the left or on the right of the fixation cross. After, the two objects disappeared and a probe (X) appeared in the position of one of the two objects (duration of the probe was 1000 ms). Participants were asked to press one key (E) if the probe was on the left and another key (I) if the probe was on the right. Attentional bias is determined by the reaction times difference between congruent trials (the probe replaces threatening stimuli) and incongruent trials (the probe replaces neutral stimuli). For individuals whose attention is systematically drawn to the threatening stimulus, reaction times are expected to be shorter (i.e., faster) for trials where the probe replaces threatening virus-related objects compared to trials where the probe that replaces neutral objects.

### Fear for COVID-19 questionnaire

The Fear for COVID-19 questionnaire is a scale specifically created to measure fear and concerning beliefs related to the COVID-19 pandemic in the Italian population^10^. The questionnaire is composed of eight items, which mainly refer to the perceived possibility of being infected by COVID-19 and to the possible consequences of the contagion. Participants are asked to answer on a scale from 0 (“not at all”) to 100 (“extremely”) how much they agree with certain statements about COVID-19. Component structure and questionnaire’s reliability were explored in a larger Italian sample (N = 4121) through principal component analysis (PCA) and Cronbach’s alpha (α). Results revealed a structure with two moderately correlated factors (r = 0.45) each composed by four items. The first factor is “Belief of contagion”, with items referring to the idea of being infected (for themselves and significant ones) by COVID-19, either in the past or in the future; the second factor is “Consequences of contagion” and items refer to the possible consequences of being infected (for themselves and others). The questionnaire’s items are reported in Table 3. For the present study, scores were computed by averaging participants’ answers on the four items of each factor.

**Table 3:** Fear for COVID-19 Questionnaire: item descriptions and pattern matrix of the PCA.

	Item
Belief of contagion	I often thought I was infected with the virus
	I think I could be infected with the virus in the future
	I think that a dear or close person to me could potentially be infected with the virus
	I think that a dear or close person to me could potentially be infected with the virus in the future
Consequences of contagion	I think that a person infected with the virus could recover
	I think that a person infected with the virus could die
	I think it is probable that I would recover after being infected with the virus
	I think that being infected with the virus could be lethal for me

### Whiteley-7 index

The Whiteley-7 Index (WI-7)^39^ is a 7-item scale, based on the Whiteley Index (WI-14)^40^. Specifically, WI-7 is composed of 7 of the original 14 items of the WI-14. Each item requires a dichotomous choice (yes or no) with a total test score ranging between 0 and 7. Higher scores indicate higher levels of health anxiety. This short-version scale has been increasingly used to assess health anxiety both in the clinical and general population as well as in the primary care context. Indeed WI-7 is a valid screening instrument with high sensitivity and specificity for hypochondriasis^40^, for DSM-IV somatization disorder^41–43^, and health anxiety in the general population^44,45^.

### Visual dot-probe data preparation

Participants were accurate for probes that replaced threatening images (93.1%) as well as for probes that replaced neutral images (93.3%). Before data analysis, visual dot-probe data were computed according to the methods described in previous studies^36^. Trials with incorrect responses were not included in the dataset and reaction times shorter than 250 ms and longer than 1000 ms, were excluded. As a result, 93.48% of the original data were included in the following analyses. Since attentional bias is indicated by shorter (i.e., faster) reaction times to congruent versus incongruent trials, each participant’s mean per trial reaction time to probes was calculated for trial type (congruent versus incongruent). No significant RT difference was found between probes that replaced virus related images (congruent trials, M = 392.01 ± 61.05 SD) and probes that replaced neutral images (incongruent trials, M = 392.76 ± 67.45 SD), [t(131) = 0.345, p = 0.73]. Finally, each participant’s attentional bias score was computed by subtracting congruent trials’ reaction times to incongruent trials’ reaction times (M = 0.74 ± 24.7 SD).

## Supplementary information

Supplementary information.

## Data Availability

The datasets generated during and/or analyzed during the current study are available in the Open Science Framework repository, https://osf.io/cswzj/?view_only=992284b06b3341818852bd9ed509fede.
