# Supplementary material for: Health anxiety and attentional bias toward virus-related stimuli during the COVID-19 pandemic
Source: Sci Rep. 2020 Oct 5;10:16476. doi: 10.1038/s41598-020-73599-8 (PMC7536432; doi:10.1038/s41598-020-73599-8)
Supplement: Supplementary file 1 — Supplementary information. [file 41598_2020_73599_MOESM1_ESM.docx]

Health anxiety and attentional bias toward virus-related stimuli during the COVID-19 pandemic

Loreta Cannito^1,⸸,*^, Adolfo Di Crosta^1,⸸^, Rocco Palumbo^2^, Irene Ceccato^1^, Stefano Anzani^1^, Pasquale La Malva^2^, Riccardo Palumbo^1^, Alberto Di Domenico^2^

^1^University G. D’Annunzio, Department of Neuroscience, Imaging and Clinical Sciences, Chieti Scalo, 66100, Italy
^2^University G. D’Annunzio, Department of Psychological Sciences, Health and Territory, Chieti Scalo, 66100, Italy

^⸸^these authors contributed equally to this work
^*^loreta.cannito@unich.it

**Table S1.** Mean score and standard deviation (S.D.) to the question: *“How much, from 0 (not at all) to 100 (very much), is this picture associated with the idea of COVID-19-?”*

| **Image Type** | **Trial** | **Description** | **Mean score** | **S.D.** |
| --- | --- | --- | --- | --- |
| Virus-related 1 | Test | Surgical mask | 98 | 10.1 |
| Virus-related 2 | Test | Disposable gloves | 95.9 | 12 |
| Virus-related 3 | Test | Laundry sanitizer | 93 | 18.5 |
| Virus-related 4 | Test | Liquid home sanitizer | 96.2 | 12.3 |
| Virus-related 5 | Test | Floor disinfectant | 89.3 | 18.4 |
| Virus-related 6 | Test | Bleach | 84.5 | 24 |
| Virus-related 7 | Test | Hand gel sanitizer | 95.6 | 11.8 |
| Virus-related 8 | Test | Hand liquid soap | 82.7 | 25.3 |
| Virus-related 9 | Test | Denatured alcohol | 92.2 | 17.2 |
| Virus-related 10 | Test | Mask FFP2 | 98.8 | 6.9 |
| Neutral 1 | Test | Spray starch | 15.2 | 23.5 |
| Neutral 2 | Test | Car spray | 6 | 12.8 |
| Neutral 3 | Test | Liquid coolant | 10.5 | 19.6 |
| Neutral 4 | Test | Shaving foam | 13.4 | 22.8 |
| Neutral 5 | Test | After shave | 12 | 19.5 |
| Neutral 6 | Test | Fertilizer | 11.7 | 19 |
| Neutral 7 | Test | Moisturizing cream | 22.5 | 28.3 |
| Neutral 8 | Test | Shoe deodorizer | 16.9 | 23.7 |
| Neutral 9 | Test | Sunscreen | 17.9 | 24.7 |
| Neutral 10 | Test | Hair spray | 13 | 21.4 |
| Filler 1 | Filler | Moka pot | 17 | 25.4 |
| Filler 2 | Filler | Steam iron | 11 | 19.2 |
| Filler 3 | Filler | Bowl | 11.6 | 19.4 |
| Filler 4 | Filler | Salt cellar | 7.1 | 13.6 |
| Filler 5 | Filler | Hair dyer | 7.6 | 14.8 |
| Filler 6 | Filler | Electric razor | 14.6 | 23.7 |
| Filler 7 | Filler | Pitcher | 10.6 | 17.6 |
| Filler 8 | Filler | Flower vase | 15.3 | 24.2 |
| Filler 9 | Filler | Jar | 12.3 | 20.6 |
| Filler 10 | Filler | Watering can | 19.1 | 26.4 |
| Filler 11 | Filler | Cooking pot | 9.3 | 17.1 |
| Filler 12 | Filler | Cooking pan | 10.2 | 17.4 |
| Filler 13 | Filler | Lunchbox | 20.9 | 27.5 |
| Filler 14 | Filler | Carafe | 6.5 | 13.4 |
| Filler 15 | Filler | Saucepan | 7.5 | 14.4 |
| Filler 16 | Filler | Casserole | 15.2 | 24.3 |
| Filler 17 | Filler | Blender | 11.7 | 20.7 |
| Filler 18 | Filler | Mixer | 15.7 | 25.2 |
| Filler 19 | Filler | Kitchen scale | 10.7 | 17.3 |
| Filler 20 | Filler | Digital kitchen scale | 22.2 | 27.8 |
